# Olive Varieties under UV-B Stress Show Distinct Responses in Terms of Antioxidant Machinery and Isoform/Activity of RubisCO

**DOI:** 10.3390/ijms222011214

**Published:** 2021-10-18

**Authors:** Chiara Piccini, Giampiero Cai, Maria Celeste Dias, Márcia Araújo, Sara Parri, Marco Romi, Claudia Faleri, Claudio Cantini

**Affiliations:** 1 Department of Life Sciences, University of Siena, Via Mattioli 4, 53100 Siena, Italy; piccini3@student.unisi.it (C.P.); sara.parri@student.unisi.it (S.P.); marco.romi@unisi.it (M.R.); faleric@unisi.it (C.F.); 2 Institute for BioEconomy, National Research Council of Italy, 58022 Follonica, Italy; claudio.cantini@ibe.cnr.it; 3 Centre for Functional Ecology, Department of Life Sciences, University of Coimbra, Calçada Martim de Freitas, 3000-456 Coimbra, Portugal; celeste.dias@uc.pt (M.C.D.); marciaaraujo@fc.up.pt (M.A.); 4 Department of Biology, Faculty of Sciences, University of Porto, Rua Campo Alegre, 4169-007 Porto, Portugal; 5 CITAB, University of Trás-os-Montes and Alto Douro, 5001-801 Vila Real, Portugal

**Keywords:** UV-B radiation, *Olea europaea*, RubisCO, antioxidant enzymes, heat shock proteins, sucrose synthase

## Abstract

In recent decades, atmospheric pollution led to a progressive reduction of the ozone layer with a consequent increase in UV-B radiation. Despite the high adaptation of olive trees to the Mediterranean environment, the progressive increase of UV-B radiation is a risk factor for olive tree cultivation. It is therefore necessary to understand how high levels of UV-B radiation affect olive plants and to identify olive varieties which are better adapted. In this study we analyzed two Italian olive varieties subjected to chronic UV-B stress. We focused on the effects of UV-B radiation on RubisCO, in terms of quantity, enzymatic activity and isoform composition. In addition, we also analyzed changes in the activity of antioxidant enzymes (SOD, CAT, GPox) to get a comprehensive picture of the antioxidant system. We also evaluated the effects of UV-B on the enzyme sucrose synthase. The overall damage at biochemical level was also assessed by analyzing changes in Hsp70, a protein triggered under stress conditions. The results of this work indicate that the varieties (Giarraffa and Olivastra Seggianese) differ significantly in the use of specific antioxidant defense systems, as well as in the activity and isoform composition of RubisCO. Combined with a different use of sucrose synthase, the overall picture shows that Giarraffa optimized the use of GPox and opted for a targeted choice of RubisCO isoforms, in addition to managing the content of sucrose synthase, thereby saving energy during critical stress points.

## 1. Introduction

The olive tree (*Olea europaea* L.) is one of the most important and oldest crops in the Mediterranean basin. Its widespread use is due to the ability to adapt to the climatic conditions typical of the Mediterranean (mild and humid winters with temperatures that rarely drop below 0 °C, hot and dry summers) [1]. Abiotic stress can have negative effects on the morphology, physiology and metabolism of olive trees and are likely among the main factors that limit olive productivity [2]. Furthermore, in recent decades, human activities have increased, through pollution of soil, water and atmosphere, and the number of potential abiotic stresses that plants must tolerate. The atmospheric pollution has led to a progressive reduction of the ozone layer with a consequent increase in UV-B radiation reaching the earth’s surface [3]. Despite the high adaption of olive trees to the environmental conditions of the Mediterranean region, the progressive increasing levels of UV-B radiation (e.g., [4,5]), together with additional environmental factors, such as sky cloudiness and high air pollutants, are a risk to olive cultivation and productivity [6,7,8]. It is therefore necessary to understand how high levels of UV-B radiation affect olive plants and to identify olive varieties better adapted to these conditions. This will allow farmers to grow selected varieties that are suitable for the current and future environmental scenarios.

It is known that intense UV-B radiation can lead to severe damage to DNA, proteins, and other cellular components [9]. Since UV-B stress has deleterious effects on proteins, heat shock protein 70 kilodaltons (Hsp70) and other chaperone proteins can play a critical role in plant defense by promoting proper refolding of denatured proteins. In fact, heat shock proteins (Hsps) not only assist in protein misfolding due to heat stress but are also involved in refolding following other types of abiotic stress, such as ultraviolet radiation [10].

One of the main targets of UV-B radiation is the photosynthetic apparatus of plants, which is highly sensitive to UV-B exposure [11]. High UV-B radiation causes a decrease in photosynthetic efficiency, reduction in the growth rate and alterations in the metabolism of carbon and nitrogen [12,13]. UV-B radiation can also affect stomatal conductance, thereby altering the net assimilation rate of CO_2_ and the rate of water loss through transpiration [11,14]. Furthermore, an excess of UV-B radiation causes inactivation of photosystem II (PSII) [11,15], a decrease in the levels of photosynthetic pigments [12,16,17], alteration of the integrity of thylakoids and changes of chloroplast ultrastructure [11], as well as reduction in RubisCO activity [6] and down-regulation of transcription of photosynthetic genes [18]. In particular, RubisCO (the enzyme that catalyzes the carboxylation step in the Calvin cycle) has been shown to be a target molecule for various stresses, such as drought and heat [19,20]. Like other proteins, it can also be damaged by reactive oxygen species (ROS), the latter being produced following exposure of plants to UV-B. Fedina et al. [21] showed that treatment with UV-B radiation on three different rice cultivars increased the activity of antioxidant enzymes, in connection with the reduction of RubisCO subunits. Other studies demonstrate that UV-B stress leads to a decrease in both the enzymatic activity and quantity of RubisCO in various plant species [6,22,23,24]. RubisCO is also characterized by many post-translational modification sites [25]; therefore, it can be speculated that a stress treatment can generate RubisCO isoforms more suitable to face stressful conditions.

High levels of UV-B radiation are known to induce in plants abundant production of ROS [26,27,28,29]. Therefore, plants have developed protective mechanisms against ROS, such as batteries of antioxidant enzymes and accumulation of UV-absorbing compounds [21,30]. Antioxidant enzymes include superoxide dismutase (SOD), catalase (CAT) and glutathione peroxidase (GPox), while non-enzymatic substances include glutathione, ascorbate, tocopherols, carotenoids, albumin, bilirubin, chelating agents and phenolics [15,31,32]. With regards to phenolic compounds, flavonoids can effectively absorb UV-B radiation and neutralize ROS [33]. Furthermore, exposure to UV-B radiation increases the concentration of other phenolic compounds that can efficiently protect plants against UV-B stress [7]. In a previous study, we showed that phenolic compounds are involved in the response of olive trees to an excess of UV-B radiation [12].

While RubisCO fuels the Calvin cycle, thus generating substrates for sucrose synthesis, sucrose degradation in sink tissues is carried out by enzymes such as invertase and sucrose synthase. Specifically, sucrose synthase catalyzes the reversible conversion of sucrose and UDP into fructose and UDP-glucose [34]. Several experimental evidence indicate that UV-B stress can affect the activity of sucrose-metabolizing enzymes, including sucrose synthase [35,36]. In combination with damage to RubisCO, the altered enzymatic activity of sucrose synthase can lead to an incorrect metabolization of sucrose, resulting in a drop in available sugars.

In this study, we integrated the contribution of previous investigations by focusing on biochemical and enzymatic analysis [12]. In particular, we analyzed two Italian varieties of *Olea europaea* (Olivastra Seggianese and Giarraffa) subjected to chronic UV-B stress (14 h per day for eight weeks). We selected the two cultivars on the basis of historical information about their long-term presence and therefore acclimatization to two very different environments in Italy. Olivastra Seggianese is a variety widespread only in its area of origin; it is mainly found around Seggiano, Tuscany, central Italy, located 490 m above sea level, with an average annual temperature around 12 °C and annual solar radiation of 170 MJ per square meter. Giarraffa, on the other hand, is cultivated in many areas of Sicily, but is also found in Calabria and Puglia; it is one of the oldest cultivars in Sicily, in the extreme south of Italy and partially southernmost off the coast of Africa, with an average annual temperature above 20 °C and solar radiation ≥ 200 MJ per square meter (http://clima.meteoam.it/ last accession 5 October 2021). We focused on the effects of UV-B radiation on RubisCO, in terms of quantity, enzymatic activity and isoform variation. Furthermore, observations by transmission electron microscopy (TEM) were performed on leaf samples to find correlations between changes in photosynthetic parameters and ultrastructural changes. In addition, we also analyzed the activity of antioxidant enzymes (SOD, CAT, GPox) to get a comprehensive picture of the antioxidant system in olive plants subjected to UV-B stress. Given the importance of sucrose, we also evaluated the effects of UV-B on the enzyme sucrose synthase. The overall damage at biochemical level was assessed by analyzing changes in Hsp70, a protein whose content is triggered under stress conditions [37].

## 2. Results

### 2.1. Microscopy Analysis

**Figure 1:**
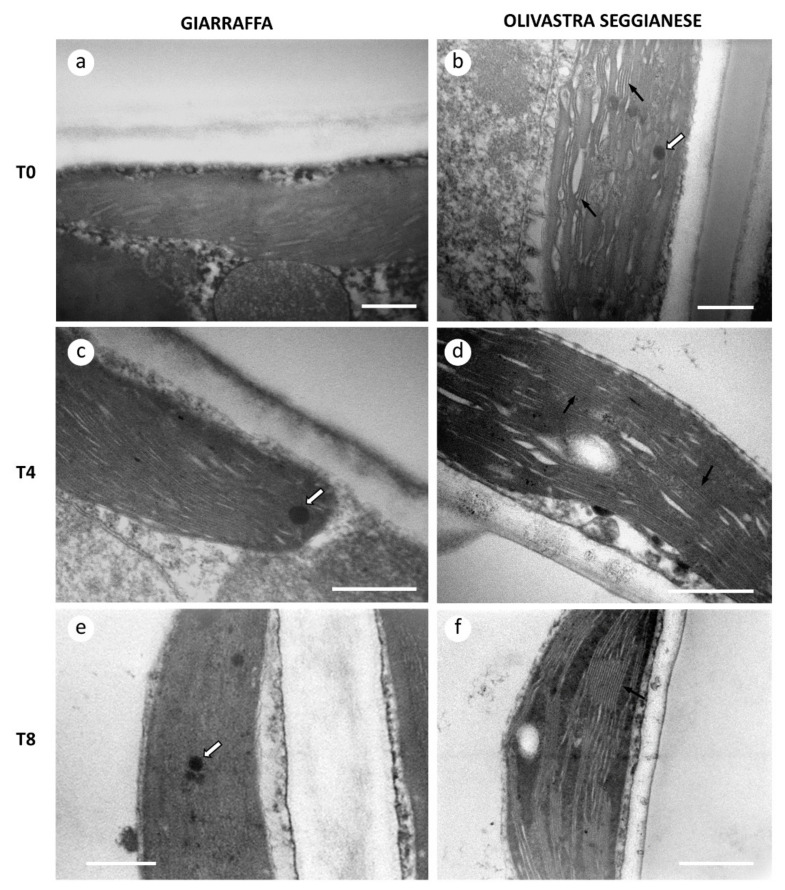
Ultrastructural analysis of chloroplasts of Giarraffa and Olivastra Seggianese leaves. (**a**) Chloroplast in Giarraffa leaves at T0; note the relative compactness of thylakoids. (**b**) Chloroplast in Olivastra Seggianese leaves at T0; thylakoids are more spaced and less compact. (**c**) Chloroplast of Giarraffa at T4, still characterized by a high compactness of thylakoids. (**d**) Chloroplast of Olivastra Seggianese at T4, characterized by a lower compactness of thylakoids. (**e**) Two chloroplasts of Giarraffa at T8, where it is still difficult to distinguish individual thylakoids. (**f**) Ultrastructure of chloroplast of Olivastra Seggianese at T8, with easily distinguishable thylakoids and grana. Black arrows indicate thylakoids, while white arrows indicate lipid bodies. Bars: 500 nm.

In the present study, observations by transmission electron microscopy (TEM) were performed on leaf samples (control and stressed) of both varieties at T0, T4, and T8. The aim was to examine whether any alterations in photosynthetic parameters (such as the amount and composition of RubisCO) could have a correlation with ultrastructural changes (Figure 1). At T0, it was readily possible to detect fundamental differences in chloroplast structure between the two varieties. In particular, Giarraffa showed a higher relative compactness of thylakoids (Figure 1a), so that it was not even easy to distinguish individual grana. Such compactness was not present in Olivastra Seggianese (Figure 1b), where the single thylakoids were clearly distinct (black arrows). Olivastra Seggianese showed the presence of some lipid bodies (white arrow), rarely observed in Giarraffa. At T4, the compactness of thylakoids in Giarraffa was maintained (Figure 1c); the presence of some small lipid bodies could be observed (white arrow). In Olivastra Seggianese at T4 (Figure 1d), individual thylakoids were still clearly discernible and well-aligned with each other (black arrows). At T8, Giarraffa chloroplasts were still characterized by a remarkable compactness of thylakoids (Figure 1e) and by the presence of sporadic lipid bodies (white arrow); in Olivastra Seggianese thylakoids and grana were still easily distinguished (black arrow). In any case, no particular ultrastructural damage was observed.

### 2.2. Antioxidant Enzymes Analysis

The ANOVA test showed a significant effect of UV-B stress on olive variety, treatment, and of their interaction on RubisCO and Gpox content (*p* ≤ 0.001), while MDA and SOD activities showed significant effect of treatment and treatment x variety (*p* ≤ 0.001). Finally, CAT activity was affected only by the specific variety (*p* ≤ 0.001) and by the interaction treatment x variety (*p* ≤ 0.005).

#### 2.2.1. Superoxide Dismutase (SOD)

**Figure 2:**
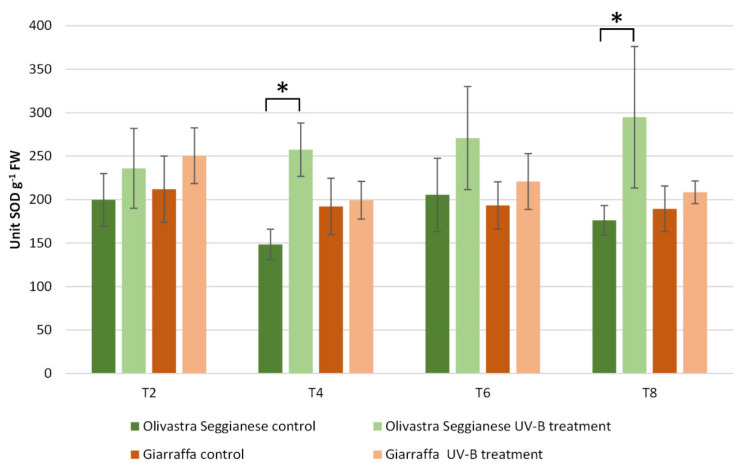
Superoxide dismutase activity in Giarraffa and Olivastra Seggianese leaves under control conditions and after exposure to UV-B treatment. The *X*-axis reports the treatment times. The asterisks (*) indicate statistically significant differences between control and stressed samples within each variety. Values are mean ± standard (*n* = 6).

The enzymatic activity assay of superoxide dismutase (SOD) (Figure 2) revealed the absence of significant differences between control and stressed plants of both varieties at T2 (*p* > 0.05). The same finding was also observed at T0 and has been omitted in this graph. After four weeks of stress (T4), a significant increase (*p* ≤ 0.01) in SOD enzymatic activity was observed in stressed Olivastra Seggianese plants compared to control plants. In contrast, Giarraffa did not exhibit any change as the stressed plants were characterized by similar SOD values to the control plants. After additional two weeks of stress (T6), the difference previously observed for Olivastra Seggianese was not present and all plants (control and stressed) of both varieties showed very similar SOD values. At the end of experiment (T8), Olivastra Seggianese again showed a significant (*p* ≤ 0.01) increase in SOD enzyme activity in treated plants compared to control plants. Giarraffa, on the other hand, did not exhibit any variation between stressed and control plants.

#### 2.2.2. Catalase (CAT)

**Figure 3:**
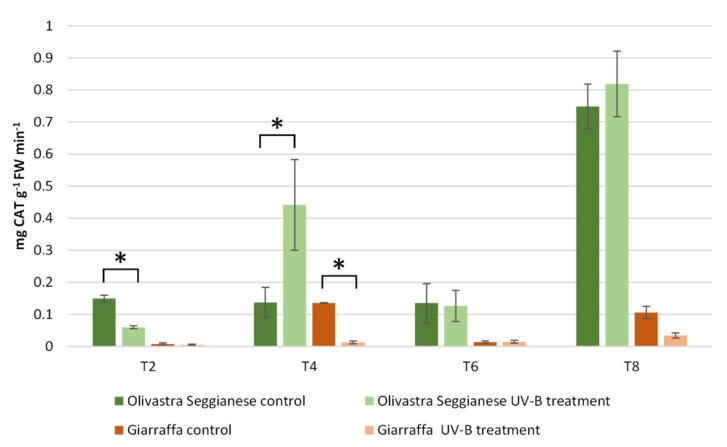
Catalase activity in Giarraffa and Olivastra Seggianese leaves under control conditions and after exposure to UV-B treatment. The *X*-axis indicates the treatment times. The asterisks (*) indicate statistically significant differences between control and stressed samples within each variety. The two varieties differ by ANOVA test for *p* ≤ 0.01. Values are mean ± standard (*n* = 6).

Enzymatic activity assay of catalase (CAT) (Figure 3) exhibited a significant difference in control plants of the two varieties (*p* ≤ 0.01) as they progressed from initial (T2) to final (T8) treatment. Basal differences in CAT enzyme activity between control plants of Olivastra Seggianese and Giarraffa were already evident at T0 (data not shown). Indeed, control plants (but also stressed plants) of Olivastra Seggianese variety showed significantly higher levels of CAT enzyme activity than plants of Giarraffa variety, with a very important increase at T8. The Giarraffa variety did not exhibit statistically significant differences (*p* > 0.05) in CAT activity between control and stressed plants throughout the experiment (from T2 to T8) with the sole exception of control plants at T4. In contrast, statistically significant differences (*p* ≤ 0.01) in CAT enzyme activity were observed between control and stressed plants of Olivastra Seggianese variety at both T2 and T4. In fact, while T2 was characterized by an increase in CAT activity in control plants, T4 conversely showed a significant increase of CAT activity in stressed plants compared to controls.

#### 2.2.3. Glutathione Peroxidase (GPox)

**Figure 4:**
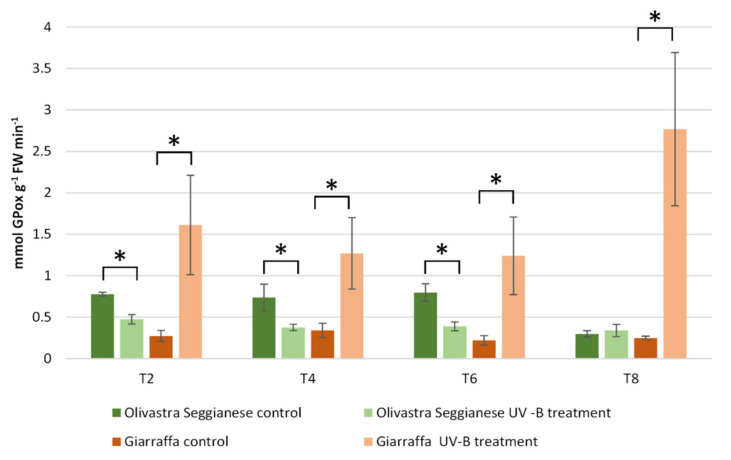
Glutathione peroxidase activity in Giarraffa and Olivastra Seggianese leaves under control conditions and after exposure to UV-B treatment. The *X*-axis indicates the treatment times. Asterisks (*) indicate statistically significant differences between control and stressed samples within each variety. The two varieties differ by ANOVA test for *p* ≤ 0.01. Values are mean ± standard (*n* = 6).

The enzymatic activity assay of glutathione peroxidase (GPox) (Figure 4) showed a statistically significant difference (*p* ≤ 0.01) in control plants of the two varieties from T2 to T8, as well as at T0 (data not shown). This difference was higher in the Olivastra Seggianese control plants than in the Giarraffa controls. For the Olivastra Seggianese variety, a significant (*p* ≤ 0.01) and stable decrease in GPox activity was observed in treated plants compared to control plants from T2 to T6. On the contrary, the Giarraffa variety showed a significant (*p* ≤ 0.01) and progressive increase in GPox enzymatic activity from T2 to T8 in stressed plants compared to control plants. Ultimately, Olivastra Seggianese plants showed a decrease in enzymatic activity after UV-B stress (except at T8) while, on the contrary, stressed Giarraffa plants showed a significant increase in GPox activity from T2 onwards.

### 2.3. Lipid Peroxidation Analysis (Malondialdehyde)

**Figure 5:**
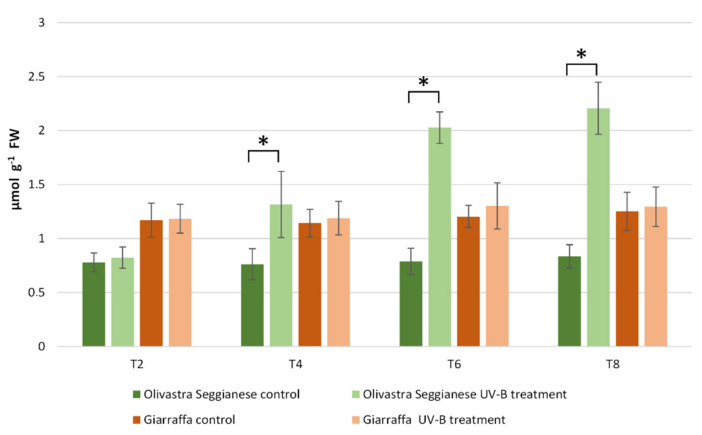
MDA (malondialdehyde) content in Giarraffa and Olivastra Seggianese leaves under control conditions and after exposure to UV-B treatment. Treatment times are indicated in the *X*-axis. The asterisks (*) indicate statistically significant differences between control and stressed samples within each variety. The two varieties differ by ANOVA test for *p* ≤ 0.01. Values are mean ± standard (*n* = 6).

Analysis of lipid peroxidation (Figure 5), as measured by malondialdehyde (MDA) production, showed a statistically significant difference (*p* ≤ 0.01) in MDA production between control plants of the two varieties from T2 to T8 (values at T0 were very similar to T2). This difference was more prominent in Giarraffa control plants than in Olivastra Seggianese controls. In addition, when examining the enzyme profile of the Giarraffa variety, no statistically significant differences (*p* > 0.05) in MDA production were shown between control and stressed plants, from T2 to T8. In contrast, statistically significant differences (*p* ≤ 0.01) were observed between control and stressed plants of the Olivastra Seggianese variety. In particular, a significant (*p* ≤ 0.01) and progressive increase in MDA production was observed in stressed plants compared with control plants from T4 to T8.

### 2.4. Ribulose-1,5-Bisphosphate Carboxylase/Oxygenase (RubisCO) Activity

**Figure 6:**
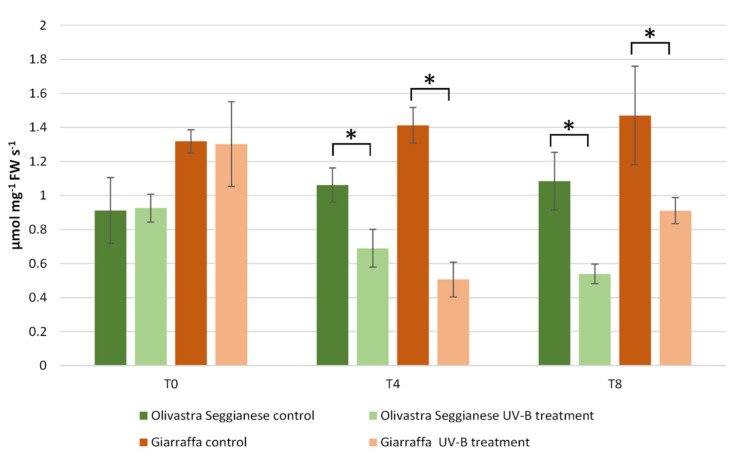
RubisCO activity in Giarraffa and Olivastra Seggianese leaves under control conditions and after exposure to UV-B treatment. The *X*-axis indicates the treatment times. The asterisks (*) indicate statistically significant differences between control and stressed samples within each variety. The two varieties differ by ANOVA test for *p* ≤ 0.01. Values are mean ± standard (*n* = 3).

The assay of RubisCO enzymatic activity (Figure 6) showed that a statistically significant difference (*p* ≤ 0.01) already occurred at T0 between the two varieties. Indeed, plants of the Giarraffa variety showed a higher RubisCO activity than plants of the Olivastra Seggianese variety. Data after the UV-B treatment indicated that radiation stress determined a significant change (*p* ≤ 0.01) in the enzymatic activity of both varieties. In particular, a significant decrease in RubisCO enzyme activity was observed at T4 in UV-B stressed plants of both varieties compared to control plants. The decrease was significantly pronounced (more than 50%) when comparing the stressed and control plants of Giarraffa to the corresponding ones of Olivastra Seggianese. At T8, stressed plants of Olivastra Seggianese were characterized by a further significant decrease in RubisCO activity compared to control plants. On the other hand, stressed plants of the Giarraffa variety exhibited a significant increase in RubisCO activity compared to T4, although with values significantly lower (*p* ≤ 0.01) than those observed in control plants at T8.

### 2.5. Proteomic Analysis

#### 2.5.1. 1-D Analysis

Protein samples extracted from control and stressed plants of both olive varieties were analyzed by one-dimensional electrophoresis to detect protein changes after UV-B stress. One-dimensional electrophoretic analysis showed no particular differences between individual varieties and between the various stages of treatment. Immunoblotting analysis was therefore performed to detect changes in the levels of specific protein such as Hsp70, RubisCO and sucrose synthase. The three proteins have been analyzed using antibodies already extensively tested in our laboratory not only on the olive tree but also on other plant species. The accumulation of the three proteins was studied in leaf samples of olive trees at T0, T4 and T8. As a preliminary remark, it should be specified that unstressed plants behaved very consistently during the UV-B treatment period, at least with regard to the levels of proteins under study. For this reason, the blots show only the comparison with the sample at T0.

##### Hsp70

**Figure 7:**
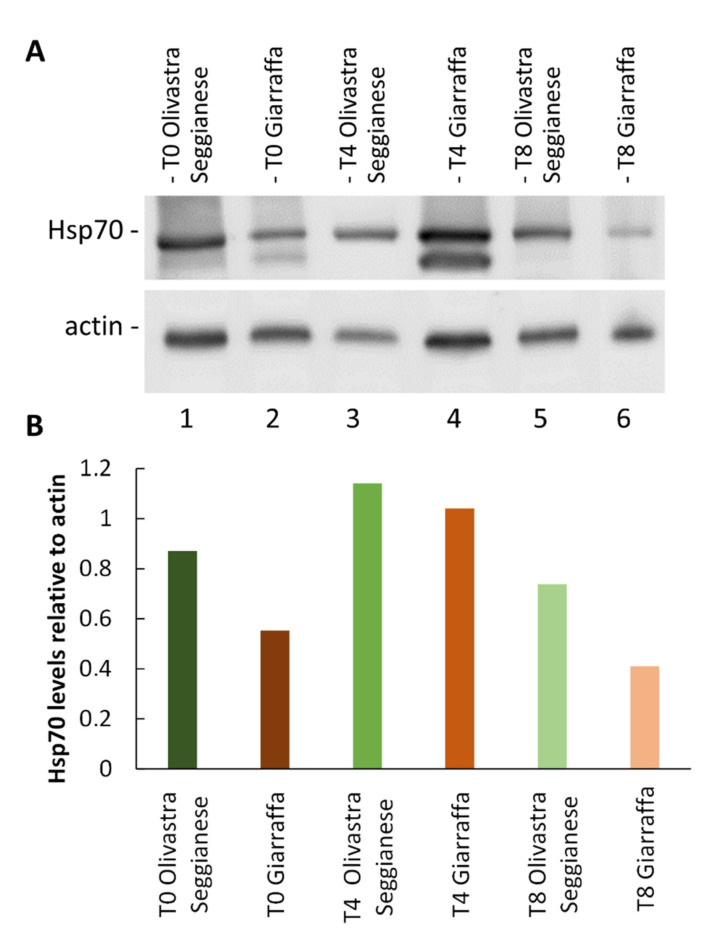
Analysis of Hsp70 content. (**A**) Electrophoresis and immunoblotting with anti-Hsp70 and anti-actin antibodies on proteins extracted from Giarraffa and Olivastra Seggianese plants, subjected to UV-B stress and collected at three selected time points (T0, T4, and T8). Lane 1: Olivastra Seggianese at T0. Lane 2: Giarraffa at T0. Lane 3: Olivastra Seggianese at T4. Lane 4: Giarraffa at T4. Lane 5: Olivastra Seggianese at T8. Lane 6: Giarraffa at T8. The same protein quantities were loaded in each lane. (**B**) Graph of the relative quantification of immunoblot intensities for Hsp70 relative to the actin content.

The results obtained from the immunoblotting analysis of Hsp70 (Figure 7) shows an increase of protein levels in both varieties at T4 compared to the control at T0. The increase is more evident in the stressed samples of Giarraffa. Subsequently, as stress progresses, we found a decrease in protein content at T8 for both varieties as compared to values recorded at T4. The decrease is more marked in stressed samples of Giarraffa than in Olivastra Seggianese. The graph in Figure 7B shows the relative intensity of immunoblotting against Hsp70 compared to the intensity of actin, the latter considered as a reference protein. It can be observed that the two varieties under consideration have distinct levels of Hsp70 at T0. However, both varieties react to stressful conditions by increasing Hsp70 levels at T4. The Giarraffa variety almost doubles the levels of Hsp70. At T8, both varieties show levels of Hsp70 comparable to control values. This means that, after an intermediate stage of protein accumulation because of UV-B stress, plants do not need higher levels of Hsp70 at later stages of stress.

##### Ribulose-1,5-Bisphosphate Carboxylase/Oxygenase (RubisCO)

**Figure 8:**
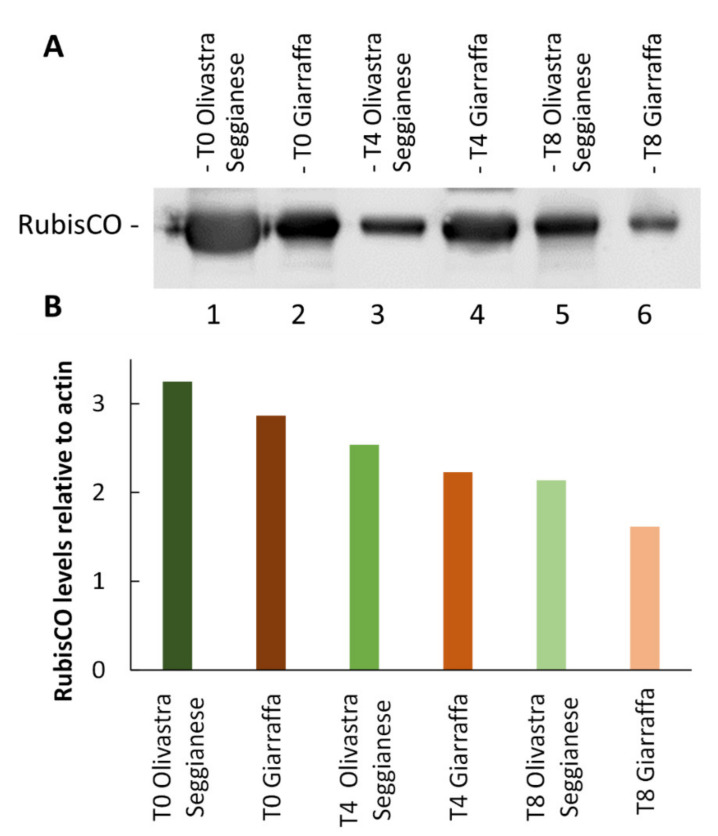
Analysis of RubisCO content. (**A**) Electrophoresis and immunoblotting with anti-RubisCO antibody on proteins extracted from Giarraffa and Olivastra Seggianese plants, subjected to UV-B stress and collected at T0, T4, and T8. Lane 1: Olivastra Seggianese at T0. Lane 2: Giarraffa at T0. Lane 3: Olivastra Seggianese at T4. Lane 4: Giarraffa at T4. Lane 5: Olivastra Seggianese at T8. Lane 6: Giarraffa at T8. (**B**) Graph of the relative quantification of immunoblot intensities for RubisCO relative to the actin content.

The results obtained from RubisCO immunoblotting analysis are shown in Figure 8A. The graph in Figure 8B was obtained by correlating the intensity of RubisCO immunoblotting against the actin blot (taken as reference protein). Both varieties have the highest RubisCO values at T0 and are characterized by a decrease in RubisCO content as UV-B treatment progresses. The decrement is very linear, and the two varieties do not differ in this parameter. It therefore appears that the RubisCO enzyme is equally sensitive to UV-B in the two varieties considered.

##### Sucrose Synthase

**Figure 9:**
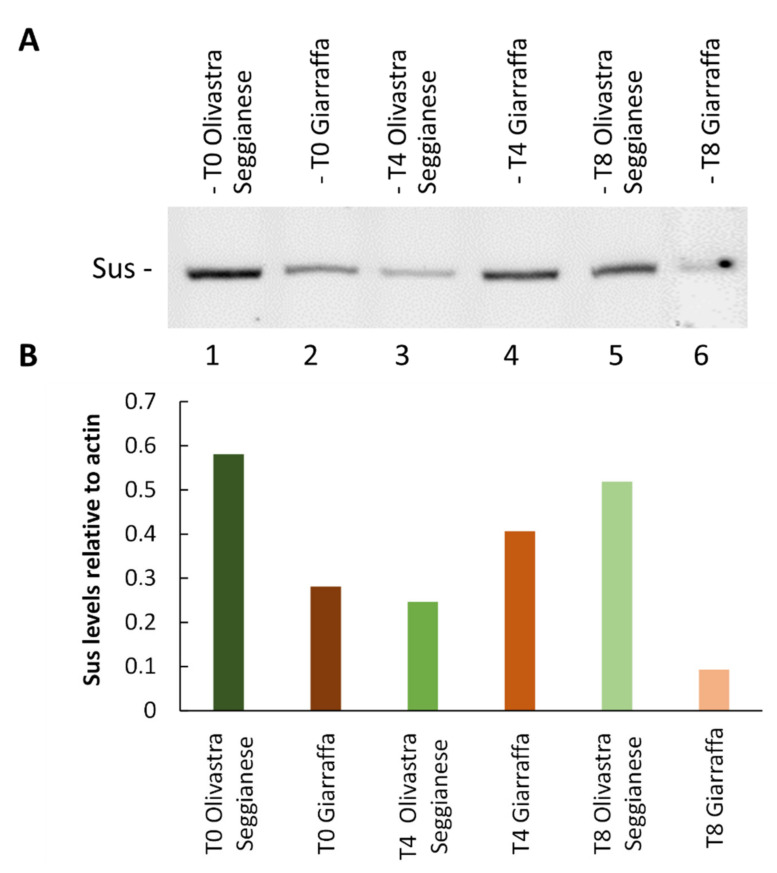
Analysis of sucrose synthase content. (**A**) Electrophoresis and immunoblotting with anti-sucrose synthase antibody on proteins extracted from Giarraffa and Olivastra Seggianese plants, subjected to UV-B stress and collected at T0, T4, and T8. Lane 1: Olivastra Seggianese at T0. Lane 2: Giarraffa at T0. Lane 3: Olivastra Seggianese at T4. Lane 4: Giarraffa at T4. Lane 5: Olivastra Seggianese at T8. Lane 6: Giarraffa at T8. (**B**) Graph of the relative quantification of immunoblot intensities for sucrose synthase relative to the actin content.

The results obtained from sucrose synthase immunoblotting analysis are shown in Figure 9A. The graph in Figure 9B was obtained by correlating the intensity of sucrose synthase blot against actin blot (taken as reference protein). The graph shows a completely opposite trend in sucrose synthase accumulation for the two varieties. In fact, the Olivastra Seggianese variety shows a significant decrease in the accumulation of sucrose synthase from T0 to T4; from T4 to T8, the enzyme content increases again, almost reaching the level of controls. In contrast, the Giarraffa variety showed a moderate increase in sucrose synthase from T0 to T4, whereas the enzyme content decreased from T4 to T8. The most striking result is the different amount of sucrose synthase at T0 between the two varieties, with Olivastra Seggianese exhibiting about twice as much sucrose synthase content as Giarraffa. The second striking result concerns the last time of analysis, T8, in which Olivastra Seggianese is able to recover the content of sucrose synthase to values comparable to controls; conversely, in Giarraffa, the quantity of sucrose synthase decreases significantly, almost to half compared to T0.

#### 2.5.2. 2-D Analysis of Ribulose-1,5-Bisphosphate Carboxylase/Oxygenase (RubisCO)

**Figure 10:**
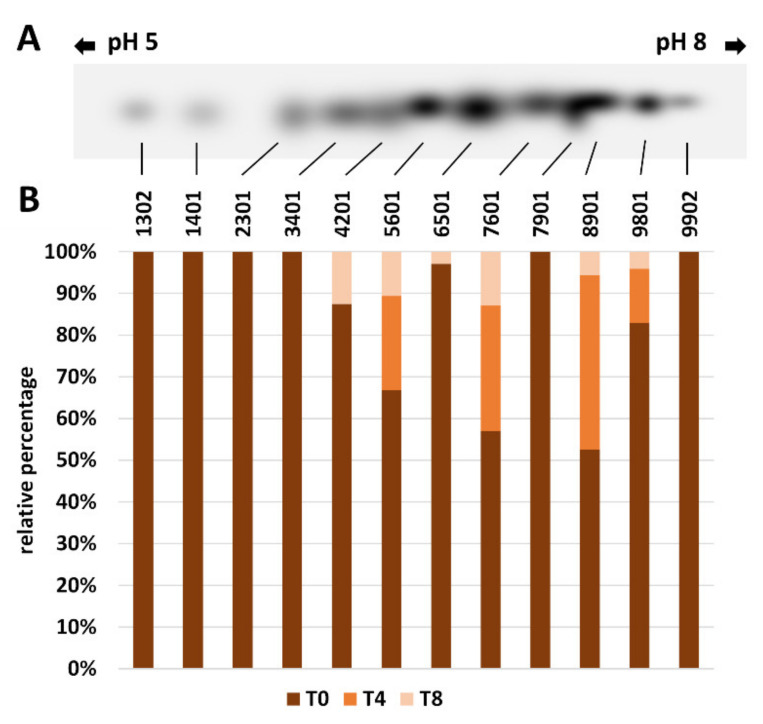
2-D analysis of RubisCO in Giarraffa plants. (**A**) Master blot of RubisCO isoforms at T0, T4 and T8 of UV-B stressed plants of Giarraffa. Each spot is identified with a numerical code. (**B**) Graph of the relative quantification of immunoblot intensities for each spot. Each spot is indicated in percentage relative to each individual analysis time.

**Figure 11:**
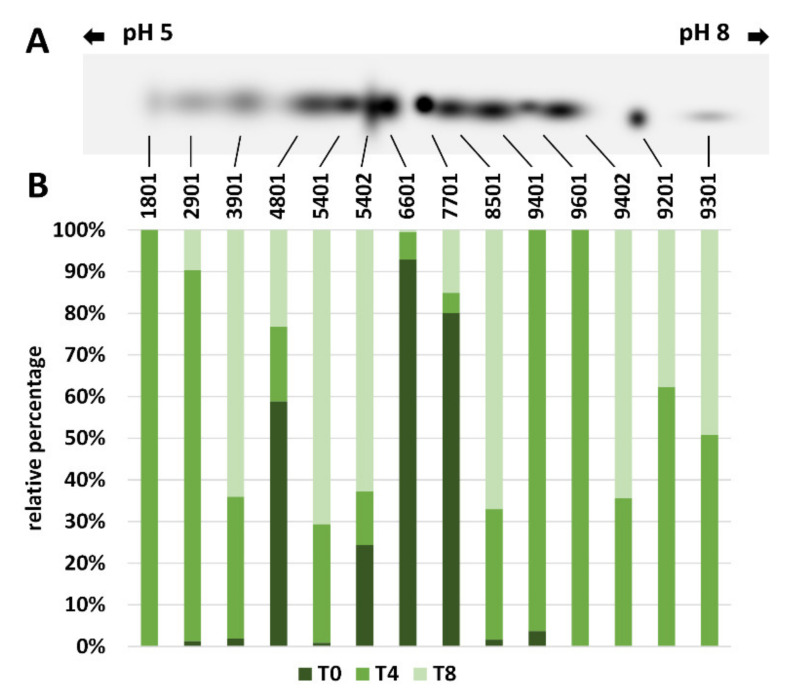
2-D analysis of RubisCO in Olivastra Seggianese plants. (**A**) Master blot of RubisCO isoforms at T0, T4 and T8 of UV-B stressed plants of Olivastra Seggianese. Each spot is identified with a numerical code. (**B**) Graph of the relative quantification of immunoblot intensities for each spot. Each spot is indicated in percentage relative to each individual analysis time.

One-dimensional electrophoretic analysis had previously shown a steady decrease in RubisCO content in both varieties. Since it is reported in the literature that RubisCO can exist in different isoforms, we analyzed whether the variation in the total RubisCO content was due to some specific isoform. For both olive varieties, two-dimensional electrophoresis and immunoblotting analysis of RubisCO were carried out at T0, T4, and T8. For both varieties, data obtained at each time point were used to construct a “master blot” containing all the RubisCO spots. The intensity of spots was then plotted for Giarraffa (Figure 10A,B) and for Olivastra Seggianese (Figure 11A,B). Starting from the master blot, the QuantityOne software associated an identification code to each of the spots identified at T0, T4 and T8. Each single spot was then compared as a percentage to the intensity of the same spot as detected in the other analysis times. This made it easier to visualize the relative intensity of each individual RubisCO isoform.

The Giarraffa variety presents clear and easily identifiable variations (Figure 10). Twelve spots could be detected at T0, but only four spots were found at T4 and six spots were identified at T8. Therefore, we noticed a consistent decrease in the number of spots because most of them were lost at T4. The RubisCO spots detected at T8 were less intense than the corresponding spots at T0. Still, at T8, the remaining isoforms were more focused in the basic region of blots, except for isoform 7901, which was present only at T0.

The Olivastra Seggianese variety, on the other hand, showed isoform variations of more complex interpretation (Figure 11). The master blot contained 10 spots at T0, 14 spots at T4 and 11 spots at T8. Of the 10 spots found at T0, only three of them (4801, 6601 and 7701) had a consistent intensity, with the others present in lesser quantities. After four weeks of treatment, the largest number of isoforms was found; however, the isoforms in the central blot area are poorly represented as compared to the more acidic and more basic spots. At the end of experiment (T8), we found that some isoforms disappeared (1801, 9401, 9601), while others showed a higher intensity than the corresponding spots detected at T4. While Giarraffa seems to focus particularly on some RubisCO isoforms during treatment from T0 to T8, Olivastra Seggianese seems to experience as many isoforms as possible without a specific selection.

## 3. Discussion

In this manuscript we have analyzed the effects of UV-B radiation on the enzymatic activity and isoform composition of RubisCO, together with the effects of UV-B on the enzyme-based antioxidant system and on the activity of sucrose synthase, one of the key enzymes in sucrose metabolism. The data obtained suggest that the two olive varieties (Olivastra Seggianese and Giarraffa) exhibit different behaviors both in terms of antioxidant response and differential use of RubisCO.

As a general stress parameter, we also analyzed the differential accumulation of Hsp70, one of the most abundant families of chaperonins involved in stress response [37]. The Hsp70 family comprises several isoforms, some of which are constitutively expressed under normal conditions as involved in cell homeostasis [38]. Results indicate an increase in Hsp70 in both varieties at T4, most evident in Giarraffa. Progression of stress results in a decrease in protein content at T8 for both varieties, most evident in Giarraffa. The increase in Hsp70 at T4 in both varieties indicates that plants suffer a stress condition after four weeks of UV-B radiation. This is not surprising because literature reports that Hsp70 are the proteins par excellence most representative of stress conditions [39]. In organisms under stressful treatments, Hsp70s are subjected to positive regulation and consequently overexpressed proportionally to stress intensity [37]. As clear proof of this, several works report that Hsp70 increases in response to abiotic stresses, such as in Arabidopsis where heat shock proteins and heat shock factors are upregulated in response to pathogen infection and abiotic stress, including UV [40]. Likewise, in soybean, Hsp70 is upregulated under high temperature stress [41], as well as in response to UV-B stress [42]. The evidence that the increase in Hsp70 coincides with the intermediate time of UV-B stress suggests that the two varieties subsequently adapt to stress conditions, especially Giarraffa, in which the content of Hsp70 decreases significantly.

Excessive UV-B radiation may increase the levels of ROS in plant cells, causing oxidative stress [43]. Targets of ROS are essential cellular components and structural elements, and accumulation of ROS is associated with lipid peroxidation, making cell membranes particularly susceptible to oxidative damage [44]. In the present study, observations by transmission electron microscopy were performed on leaf samples (control and stressed) of both varieties. At T0, we found fundamental differences in chloroplast structure between the two varieties, with Giarraffa showing a higher relative compactness of thylakoids, which was maintained at T4 and T8. Such compactness, instead, was not present in Olivastra Seggianese, at T0, T4 and T8. Thylakoid membranes are particularly sensitive to ROS. Therefore, damages on thylakoid membranes can result in reduced photosynthetic activity. A decrease in photosynthetic efficiency in olive trees subjected to UV-B stress has already been observed in our previous work [12]; reduction of photosynthetic activity was found in both varieties but with important differences. In fact, the Giarraffa variety was not able to immediately preserve the photosynthetic efficiency but an adaptation-triggered stress protective mechanism allowed the UV-B stressed plants to re-establish photosynthetic performance. The Olivastra Seggianese, on the other hand, responded earlier but was not able to maintain this capacity over time. In the present work we analyzed MDA as a parameter of ROS induced oxidation in macromolecules (namely lipids). Giarraffa showed no statistically significant differences in MDA production. In contrast, differences were observed in Olivastra Seggianese, specifically an increase in MDA production from T2 to T8. The absence of significant changes in MDA production in stressed plants of Giarraffa agrees with our previous results of photosynthetic efficiency [12] and suggests that the Giarraffa variety shows tolerance to UV-B conditions. These results are in line with what previously shown for the “Galega Vulgar” variety [6], where the UV-B treatment did not increase lipid peroxidation. It should be noted, however, that plants of the Galega Vulgar variety were exposed to a lower amount of UV-B radiation and for a shorter exposure time. This suggests again that plants of the Giarraffa variety, in contrast to the Olivastra Seggianese variety, better tolerate the UV-B stress. The mechanism underlying the improved tolerance could involve the increase in Hsp. As mentioned above, the Hsp family acts as the first defense line against heat stress in olive plants [45,46,47], as well as against other abiotic stresses such as UV [40,42]. Our hypothesis is that the increase of Hsp70 levels at T4 in stressed plants of Giarraffa may justify the absence of lipid peroxidation in stressed Giarraffa plants.

To cope with UV-B exposure, as well as to help maintain ROS levels and avoid oxidative damage, plants can activate additional mechanisms. The main defense mechanism against ROS and oxidative stress is the antioxidant defense system. Antioxidants include enzymes like superoxide dismutase (SOD), catalase (CAT) and glutathione peroxidase (GPox), as well as non-enzymatic molecules like ascorbate, tocopherols, carotenoids, albumin, bilirubin, chelating agents and phenolics [7,15,31]. In our previous work [12], we analyzed the changes in phenolic content, especially polyphenols and flavonoids. The profile of total polyphenols showed considerable difference already at T0 between the two olive varieties. Giarraffa responded after just the first week to UV-B radiation by increasing the pool of polyphenols. On the other hand, plants of Olivastra Seggianese responded later to UV-B by triggering an increase of polyphenols only at T2. In addition, the analysis of flavonoids indicated that Giarraffa still responded earlier to UV-B stress (during the first week), and total flavonoid levels decreased over time. On the contrary, Olivastra Seggianese responded later (after the second week) and maintained high levels of these compounds until the end of treatment. These distinct profiles of UV-B triggered-antioxidant response support the hypothesis that Giarraffa activates defense mechanisms already after the first week of UV-B stress, thereby performing better than Olivastra Seggianese in the long term. This improved defense capacity of Giarraffa is also supported by the slight decrease of antioxidants over the second week, which may result from its efficient neutralization of ROS, leading to an enhanced protection of olive plants from oxidative damage [7]. To complement the previous results, here we analyzed antioxidant enzymes such as SOD, CAT, and GPox. In brief, SOD enzyme activity in Giarraffa showed no variation between stressed and control plants; in contrast, stressed Olivastra Seggianese plants showed a significant increase at T4. Giarraffa showed no statistically significant differences in CAT activity while differences in CAT activity were observed in Olivastra Seggianese at T2 and T4. In addition, Olivastra Seggianese plants showed a decrease in GPox activity while stressed Giarraffa plants showed a significant increase in GPox activity from T2 onward. This suggests that the response of stressed Olivastra Seggianese plants was based on stimulation of SOD activity to convert increased O_2_^•^^-^ in H_2_O_2_, which is immediately scavenged by the stimulated CAT activity, in particular at T4. On the contrary, stressed plants of Giarraffa invest in the GPox pathway, as they show a constant and progressive increase in enzyme activity for the duration of stress. Supporting our data, other authors demonstrated that SOD, CAT and GPox activities increased in responses to UV-B stress [7,48]; Rácz et al. [31] highlighted the importance of GPox in acclimation to enhanced UV-B radiation. *Deschampsia antarctica*, an Antarctic species well acclimated to high UV-B radiation, also showed low indications of oxidative damages and a homeostatic control of ROS due to an increase of SOD, APX, CAT and GPox activities, and of total phenolic content [49]. Profiles of antioxidant response (both enzymatic and non-enzymatic) to UV-B stress may support the hypothesis that Giarraffa appears better suited to prolonged UV-B stress, possibly because of a more efficient and quick activation of antioxidant metabolites (such as flavonoids) and of the GPox activity.

Like other proteins, RubisCO can be damaged by UV-B exposure [26,27,29]. In the present study, we analyzed the activity of RubisCO, and results showed a significant decrease in RubisCO activity at T4 in UV-B stressed plants of both varieties compared to the control. The decrease was significantly more pronounced when comparing stressed/control plants of Giarraffa with corresponding plants of Olivastra Seggianese. At T8, Olivastra Seggianese stressed plants showed a significant decrease in RubisCO activity. On the other hand, Giarraffa stressed plants showed an increase in RubisCO activity compared with T4. These results are in line with data in the literature showing that UV-B stress leads to a reduction in the enzymatic activity of RubisCO in various plant species [6,22,24]. However, these results do not fully correlate with those obtained by immunoblotting analysis of RubisCO. In that case, both varieties are characterized by a decrease in RubisCO content as UV-B treatment progresses. The reduction is extremely linear, and the two varieties do not differ in this parameter. These results indicate that RubisCO is equally sensitive to UV-B in the two varieties. Fedina et al. [21] also demonstrated that UV-B radiation induced quantitative damage to the RubisCO protein. Treatment with UV-B radiation on three different rice cultivars increased the activity of antioxidant enzymes, along with reduction of RubisCO subunits. Therefore, the quantity of RubisCO decreases comparably in the stressed plants of both varieties; however, in stressed plants of Giarraffa, the gradual decrease in protein quantity does not correspond to the gradual decrease in enzymatic activity. This suggests that stressed plants of Giarraffa implement a defense mechanism to allow plants to gradually regain the RubisCO; this could allow the Giarraffa plants to recover photosynthesis better than stressed plants of Olivastra Seggianese, which conversely show a gradual decrease in the activity and quantity of RubisCO in the course of stress. RubisCO is characterized by many potential co-/post-translational modification sites [25]; therefore, it is assumed that, following UV-B stress, modifications can generate RubisCO isoforms more suitable for coping with a stressful situation. As support for this hypothesis, two-dimensional electrophoresis and immunoblotting were performed at T0, T4, and T8 on stressed and control plants of both olive varieties. Spot analysis in Giarraffa suggested a decrease in the number and intensity of RubisCO isoforms after UV-B treatment and that only undamaged isoforms or those able to effectively function despite the stressful situation persist. This would allow stressed plants of Giarraffa to recover the enzymatic activity of RubisCO. The Olivastra Seggianese variety, on the other hand, shows variations in isoforms of more complex interpretation. Basically, only three isoforms remained constant, presumably being the most functional isoforms in the absence of stress. After four weeks of treatment, the number of isoforms increased, while at the end, some isoforms disappeared and others increased in intensity. This could suggest that Olivastra Seggianese takes longer than Giarraffa to discover more functional isoforms to be used during stress or that the best response consists of a mix of different isoforms, which are nevertheless assembled in a longer time. Therefore, we assume that stressed plants of Giarraffa react better than Olivastra Seggianese to UV-B stress by post-translationally modifying RubisCO so as to produce more effective isoforms [12].

Like all other plants, olive trees must produce sugars (such as sucrose) for growth. Previously we analyzed the changes in photosynthetic sugars under UV-B stress [12]. Results from our study showed that no significant differences were found in sucrose content between control and UV-B stressed plants of both varieties. Instead, glucose and fructose were the most responsive to UV-B treatment. UV-B stressed plants of Olivastra Seggianese accumulated less glucose, particularly after the second week, possibly due to a reduction of photosynthesis and to a higher use of glucose to maintain cellular respiration or even to increase the levels of polyols (e.g., mannitol, that increases at T2) [50,51,52]. On the other hand, UV-B seemed to promote fructose accumulation (except at T6) more significantly in Olivastra Seggianese. Increase of fructose can result from sucrose degradation as a stress response or can provide substrates for the synthesis of secondary metabolites [50]. Dias et al. [6] reported that olive plants treated with a lower UV-B dose (12 kJm^−2^ d^−1^) produced less sucrose and starch but maintained the content of glucose and sorbitol. Given the key role of sucrose [53,54,55], we assumed that plants under UV-B stress implemented mechanisms to preserve both the content of sucrose and related metabolic processes. In light of this, we have analyzed the changes in the amount of sucrose synthase (SuSy) by immunoblotting. We found a completely opposite profile of SuSy accumulation for the two varieties. Olivastra Seggianese shows an initial decrease in SuSy accumulation, while thereafter the enzyme content increases again. In contrast, Giarraffa shows an initial moderate increase in SuSy content, while the enzyme content subsequently decreases. It is striking that the initial amount of SuSy differs between the two varieties, as well as the recovery of SuSy by Olivastra Seggianese at T8, while in Giarraffa the amount of SuSy decreases significantly. SuSy reversibly catalyzes the production of fructose and UDP-glucose from sucrose [56], preserving a large part of the energy available in sucrose. Given that plants of Giarraffa increase the quantity of SuSy at T4 (when plants are more under stress), this suggests that Giarraffa plants counteract the stressful conditions by storing energy in UDP-glucose and that they do not need to use all the energy contained in the sucrose molecule. After T4, the quantity of SuSy decreases considerably up to T8, which corresponds to the time when plants of Giarraffa, unlike Olivastra Seggianese, have resumed their metabolic processes. On the contrary, plants of Olivastra Seggianese show a remarkable increase in the content of SuSy at T8 compared to T4, probably because at T8 they are still suffering a severe stress and thus require all the energy available from sucrose breakdown (likely by invertase). This is also confirmed by results from our previous work [12], for which plants need to continue splitting sucrose into glucose and fructose to counteract stress conditions.

In conclusion, the results of this work indicate that the varieties Giarraffa and Olivastra Seggianese differ significantly in the use of specific antioxidant defense systems, as well as in the activity and isoform composition of RubisCO. Combined with a different use of sucrose synthase, the overall picture shows significant biochemical differences between the two olive varieties. In particular, Giarraffa optimized the use of GPox, opted for a targeted choice of RubisCO isoforms and managed the content of SuSy, saving energy during the critical stress point. This highlights once again how the two varieties were able to adapt to different environmental conditions. The two regions in which the varieties have developed (Tuscany and Sicily) are indeed characterized by different climatic parameters (higher temperatures and drought in Sicily), as well as by probably different UV-B radiation. We therefore hypothesize that biochemical adaptations are part of the global mechanism by which the two varieties respond independently to UV-B treatment. Although preliminarily, the Giarraffa variety is better equipped to tolerate UV-B radiation.

## 4. Materials and Methods

### 4.1. Plant Growth Conditions and Application of UV-B Treatment

Olive trees (*Olea europaea* L.) of 18 months (both Olivastra Seggianese and Giarraffa varieties) were taken from the nursery of the “Società Pesciatina di Orticoltura” (Pescia, PT, Italy). Subsequently, plants were transferred to climatic cells with the following environmental conditions: temperature of 21 °C; relative humidity (RH) of 60%; photoperiod of 14 light h, 10 dark h [22]; light intensity of 500 µmol m^−2^ s^−1^; watering with 400 mL water for each plant once a week; commercial substrate type “Vigor Plant Soil” (Vigorplant Italia Srl, Fombio, Italy) [12]. Ultraviolet radiation was provided by two TL20W/12 lamps (Philips, Milano, Italy) that emit in the wavelength of UV-B rays and that have already been widely used and described in the literature; lamps were used exactly according to the protocol of Allen et al. [22]. Plants (*n* = 16 for each variety) were positioned under UV-B lamps in the climatic cell. Every day, the homogeneity of UV-B radiation emitted by lamps was verified using a Power Meter 840 with Sensor 818-UV (Newport Optical, California, USA). The UV-B biologically effective dose (BED), 25 kJm^−2^ d^−1^, was calculated according to Correia et al. [57]. Control plants (*n* = 16 for each variety), present in the same climatic cell, have been carefully separated from those treated by means of a plasterboard panel that shielded most of the UV radiation (BED of 1 kJm^−2^ d^−1^). The UV-B treatment corresponds to a high UV-B dose, but within the natural values already reported on the earth’s surface [58].

### 4.2. Antioxidant Enzymes Extraction and Quantification

Olive leaves were collected at selected time points (T2, two weeks; T4, four weeks; T6, six weeks and T8, eight weeks of treatment), immediately frozen in liquid nitrogen and stored at −80 °C. Upon use, leaves were ground (0.5 g) with 5 mL of extraction buffer containing 0.1 M potassium phosphate buffer (pH 7.5), 0.5 mM Na_2_EDTA, 2 mM DTT, 1 mM PMSF, 1% PVP (*m*/*v*) and 0.2% Triton X-100 (*v*/*v*) [7]. The lysates were centrifuged at 10,000× *g* for 15 min at 4 °C and used to determine the activities of SOD (EC1.15.1.1), CAT (EC 1.11.1.6) and GPox (EC 1.11.1.7).

For SOD activity, 50 mM potassium phosphate buffer (pH 7.8), 13 mM methionine, 50 mM Na_2_CO_3_, 0.1 M Na_2_EDTA, 25 mM NBT, and the leaf extracts were mixed. Riboflavin (2 mM) was added, and the reaction was started by illuminating (fluorescent lamp of 15 W) the samples for 15 min. The absorbance was read at 560 nm, and one unit of enzyme activity was defined as the amount of SOD necessary to induce 50% inhibition on the rate of NBT reduction [59]. CAT activity was determined at 25 °C according to Beers and Sizer [60]. The reaction mixture contained 0.1 M potassium phosphate buffer (pH 7.0) and the leaf extract. To start the reaction, 20 mM H_2_O_2_ was added and after 5 min the reaction was stopped by addition of 150 mL of H_2_SO_4_ + 1 g of TiO_2_ + 10 g of K_2_SO_4_. The mixture was centrifugated at 10,000× *g* for 10 min at 4 °C and the absorbance of supernatant was read at 415 nm. The activity of catalase was determined from a standard curve. GPox activity was determined in a mixture of 96 mM guaiacol, 12 mM H_2_O_2_, 10 mM potassium phosphate buffer (pH 6) and the leaf extract [7]. GPox activity was calculated measuring the increase of absorbance at 470 nm.

### 4.3. Lipid Peroxidation

Lipid peroxidation was determined by measuring the formation of malondialdehyde (MDA) [61]. Frozen leaves, collected at the selected time points (T2, T4, T6 and T8), were ground (100 mg) with 1.5 mL of 0.1% trichloroacetic acid (TCA, *w*/*v*) and centrifugated at 10,000 g for 5 min at 4 °C. Then, 1 mL of the supernatant was homogenized with 1 mL of 20% TCA (*w*/*v*) + 0.5% of thiobarbituric acid (*w*/*v*) as a positive control; in parallel, 1 mL of sample was homogenized with 1 mL of 20% TCA (*w*/*v*) as a negative control. Both groups were incubated at 95 °C for 30 min, cooled on ice and centrifuged (10,000× *g* for 10 min at 4 °C). The absorbance of the supernatant was read at 600, 532 and 440 nm in a spectrophotometer. MDA equivalents were determined according to Hodges et al. [61].

### 4.4. Ribulose-1,5-Bisphosphate Carboxylase/Oxygenase (RubisCO) Activity

Olive leaf samples of both varieties were taken at 3 selected time points: before the onset of stress (T0), and after 4 weeks (T4) and 8 weeks of stress (T8). Subsequently, leaves were homogenized at 0 °C with 1 mL homogenization buffer consisting of 50 mM TRIS/HCl pH 7.8, 1 mM EDTA, 20 mM MgCl_2_, 10 mM NaHCO_3_, 5 mM DTT, 0.3 % BSA (w/v) and 10 mg/mL Polyclar AT (SERVA Electrophoresis GmbH, Heidelberg Germany). After centrifugation (9000 × g 5min), samples were incubated for 20 min at room temperature prior to analysis according to Lilley and Walker [62]. The supernatant was mixed with the reaction medium consisting of 50 mM Hepes/KOH (pH 8.0), 10 mM KCl, 1 mM EDTA, 20 mM MgCl_2_, 5 mM DTT, 2.5 mM ATP, 0.2 mM NADH, 10 mM NaHCO_3_, 5 mM creatine phosphate, 20 U/mL creatine phosphokinase, 6 U/mL phosphoglycerate kinase, 6 U/mL glyceraldehyde phosphate dehydrogenase and 10 µL of the extract. After establishing a steady base rate, the reaction was started with the addition of 0.6 mM ribulose-1,5 bisphosphate. The reaction was measured via the decrease in absorbance at 340 nm due to NADH oxidation.

### 4.5. Protein Extraction

Olive leaf samples of both varieties were taken at 3 selected time points (T0, T4, and T8). Samples were extracted according to Wu et al. [63], with a protocol effective in the extraction of proteins from recalcitrant plants such as olive and grapevine. Reagents were purchased from Sigma Aldrich (Merck Life Science S.r.l., Milano, Italy). All samples were processed simultaneously to minimize experimental variability. Protein concentration of samples was determined using the 2-D Quant kit (GE HealthCare, Cytiva Europe GmbH, Milano, Italy). The protocol was carried out exactly as described in the instruction manual using BSA as a reference. Each sample was analyzed in three replicates using a Shimadzu UV-160 spectrophotometer set at 480 nm.

### 4.6. 1-D Electrophoresis, Western Blotting and Image Analysis

Separation of proteins by 1-D electrophoresis was performed on Tris-HCl 10% gels using a Criterion cell (Bio-Rad Laboratories, Milano, Italy) equipped with a Power Pac Bio-Rad 300 at 200 V for approximately 35 min. TGS (25 mM Tris-HCl pH 8.3, 192 mM glycine and 0.1% SDS) was used as running buffer. Gels were stained with Bio-Safe Coomassie blue (Bio-Rad Laboratories, Milano, Italy). Transfer of proteins from gels to nitrocellulose membranes was performed using a Trans-Blot Turbo Transfer System (Bio-Rad) according to the manufacturer’s instructions. The quality of blotting was determined by checking the transfer of precision pre-stained molecular standards (Bio-Rad). After blotting, membranes were blocked overnight at 4 °C in 5% Blocking Agent (Bio-Rad) in TBS (20 mM Tris pH 7.5, 150 mM NaCl) plus 0.1% Tween-20. After washing with TBS, membranes were incubated with the primary antibody for 1 h at room temperature. For immunodetection of actin, we used the mouse monoclonal antibody clone 10-B3 diluted 1:3000 (Sigma Aldrich, Merck Life Science S.r.l., Milano, Italy), for immunodetection of RubisCO we used the rabbit polyclonal antibody clone AS03-037 diluted 1:3500 (Agrisera, Vännäs, SWEDEN), for immunodetection of SuSy the rabbit polyclonal antibody clone AS15-2830 diluted 1:5000 (Agrisera), and for immunodetection of Hsp70 we used the rabbit polyclonal antibody clone AS08-371 diluted 1:5000 (Agrisera). Subsequently, membranes were washed several times with TBS and then incubated for 1 h with peroxidase-conjugated secondary antibodies. Specifically, we used a goat anti-mouse IgG (Bio-Rad) and a goat anti-rabbit IgG (Bio-Rad) both diluted 1:3000. After additional washes in TBS, the “Clarity” (Bio-Rad) mixture was used for enzymatic reaction. Images of gels and blots were acquired using a Fluor-S apparatus (Bio-Rad), while analysis of gels and blots was performed with the Quantity One software (Bio-Rad, version 4.6.7). All blots were developed using identical conditions, from substrate incubation to exposure time. All images were processed correspondingly using the Autoscale command (to improve the quality of gels and blots) and the Background Subtraction command (to remove the background noise). The relative intensity of single bands was calculated with the Volume tool of Quantity One software (Bio-Rad, version 4.6.7). Results were exported and graphed with Microsoft Excel.

### 4.7. 2-D Electrophoresis, Western Blotting and Image Analysis

Separation of proteins by 2-D electrophoresis was performed on an IPG Strip (Ready Strip IPG Bio-Rad), 11 cm long. Since the isoelectric point of RubisCO is between 6 and 7, strips with a pH range of 5–8 were chosen. Strips were hydrated (overnight) in a solution containing the rehydration/solubilization buffer to which 18 mM DTT and 20 μL/mL IPG Buffer (pH 3–10) were added. Samples to be analyzed were also included in the rehydration/solubilization buffer. Rehydration took place in a special container (GE Immobiline Dry Strip Reswelling Tray) after strips were covered with Mineral Oil (Bio-Rad). Following rehydration, the first electrophoretic run was performed using the Protean IEF (Bio-Rad) system, with the following protocol:

1. From 0 to 500 V in 1 h
2. 500 V constant for 1 h
3. From 500 V to 4000 V in 2 h
4. 4000 V for 2 h
5. From 4000 V to 8000 V in 2 h
6. 8000 V constant up to 15000 V/hour
7. From 8000 V up to 500 V in 30 min
8. 500 V until the strips are taken.

At the end, strips were taken and immediately processed for separation of proteins in the second dimension. Strips were first equilibrated in 50 mM Tris-HCl pH 8.8, 6 M urea, 30% glycerol, 2% SDS, trace amounts of Bromophenol Blue, and 10 mg/mL DTT. We used Criterion XT PreCast 10% gels (Bio-Rad). The electrophoretic run was performed with the Criterion Cell (Bio-Rad) at 200 V constant for 1 h using the XT-MOPS (Bio-Rad) buffer. Subsequently, gels were processed and transferred to a nitrocellulose membrane for immunoblotting as described above. Membranes were blocked overnight at 4 °C in 5% ECL Blocking Agent (Bio-Rad) in TBS (20 mM Tris pH 7.5, 150 mM NaCl) plus 0.1% Tween-20. Membranes were incubated for 1 h at room temperature with a primary anti-RubisCO antibody, diluted 1: 10,000 (Agrisera code AS03037). After washings, membranes were incubated for 1 h with a secondary anti-rabbit antibody, diluted 1: 3000 conjugated to peroxidase. Images of gels and blots were acquired using a Bio-Rad Fluor-S Multi-Imager, controlled by Quantity One (Bio-Rad) software. For the comparison of immunoblots, the PDQuest software (Bio-Rad, version 8.0) was used, allowing for the alignment and relative quantification of spots. Immunoblots were analyzed according to the olive variety by comparing the three time points (T0, T4 and T8); the PDQuest software creates a reference image (“master blot”) by which the various spots can be aligned. Spot quantitation data were exported and graphed with Microsoft Excel. Blot analysis was repeated at least three times in samples from different experiments.

### 4.8. Microscopy Analysis

We analyzed olive leaves of both varieties taken at 3 selected time points (T0, T4, and T8). The protocol is detailed in Behr et al. [64]. For transmission electron microscopy (TEM), samples were fixed in 3% glutaraldehyde in cacodylate buffer (0.066 M, pH 7.2), for 1 h at room temperature. After fixation, samples were rinsed with cacodylate buffer and post-fixed with osmium tetroxide 1% in cacodylate buffer for 1 h. Then, samples were rinsed with water and dehydrated gradually in increasing concentrations of ethanol (from 10% to 100%). Samples were embedded in Spurr’s resin [65], polymerized for 8 h at 70 °C, and then cut into 600-Å sections using an LKB Nova ultramicrotome provided with diamond knife. Sections were stained with uranyl acetate and lead citrate for 10 min, respectively, and finally observed with a Philips Morgagni 268D transmission electron microscope operating at 80KV and equipped with a MegaView II CCd camera (Philips Electronics). Three different sets of experiments were subjected to TEM analysis.

### 4.9. Statistical Analysis

Statistical analysis was performed by the Systat 11 statistical package (Systat Software Inc., Richmond, CA, USA). Data were checked for normality distribution by the Shapiro–Wilk test before repeated measures of ANOVA analysis. ANOVA tested the significance of each of the three variables: time, treatment and cultivar, as well as their interaction. When the *p* values of the ANOVA were ≤ to 0.01 or 0.05, Tukey’s pairwise mean comparison within each variable was performed.

## Data Availability

Data available on request due to restriction.
